# Commentary: Effect of Low-Frequency Repetitive Transcranial Magnetic Stimulation on Impulse Inhibition Methamphetamine in Abstinent Methamphetamine Patients

**DOI:** 10.3389/fpsyt.2020.561246

**Published:** 2020-09-25

**Authors:** Hebin Wang, Xingxing Li, Chang Yu, Fangzhen Hu, Wenbang Liu

**Affiliations:** ^1^Department of Psychiatry, Ningbo Kangning Hospital, Ningbo, China; ^2^Department of Psychiatry, Yongkang Third Hospital, Jinhua, China

**Keywords:** transcranial magnetic stimulation, low-frequency, methamphetamine, commentary, prefrontal cortex (PFC)

Drug addiction and relapse are characterized by compulsive drug seeking behavior and high impulsivity throughout different stages of addiction (e.g., formation and relapse) (1). Methamphetamine (MA) dependents exhibited high impulsivity, which act as important risk of relapse (2). Neuroimaging studies reported dopaminergic transmission and cortical-striatal circuitry mechanisms underlying the changes (3). It is conceivable that targeting these aberrant circuits might facilitate the behavioral control and reduce behavioral impulsivity in MA dependents (4) and, finally, prevent the patients from relapse. A recent study published on *JAMA Netw Open* proved the clinical potential of brain stimulation approach for impulsivity control in MA patients (5).

The authors firstly developed a two-choice oddball task that allows for quantification of behavioral impulsivity with improved sensitivity, in compared to the classical Go/Nogo task (6). The task contains both standard (frequently) and deviant (infrequently) trials; the subject needs to habituate for standard trial responses and response for deviant trial by press the button for inhibition. The behavioral results therefore provided both accuracy responses and reaction time (RT) differences between the two conditions. The authors found that the MA dependents reported lower accuracy and shortened response time, both of which reflecting the increased impulsivity associated with MA dependence.

Considering the importance of prefrontal cortex (PFC) in behavioral inhibition and risk decision making processes (7), the authors therefore designed a randomized controlled trial to investigate the effects of 1 Hz non-invasive repetitive transcranial magnetic stimulation (rTMS) on impulsivity of MA dependents, which is found to be effective in reducing impulsivity in other types of patients (8). Single session 1-Hz treatment at left PFC successfully improved accuracy and slowed down the response time, while repeated treatment with 10 sessions demonstrated lasting effects for at least 30 days. These findings suggest that 1 Hz rTMS could reduce behavioral impulsivity and improve the cautious decision making.

The study for the first time reported the effects of rTMS intervention on impulsivity of MA dependence. It is possible that this finding might be generalize to other substances of abuse as well and warrants future large trials investigating the effects of chronic rTMS on impulsive drug seeking behaviors and relapse. Recent studies emphasized the potency of rTMS treatment on drug intake behavior, improve sleep quality and cognition, and efficiency to reduced cue reactivity or craving in a variety of drug dependents (9–17). It should be noted that most of these results focused on high-frequency (e.g., 10 Hz) rTMS stimulation at left PFC region (18).

Previous studies reported that, for MA addicts, the craving rate decreased after high-frequency (10 Hz) rTMS at left PFC, and the rate increased after low-frequency (1 Hz) rTMS at the same cortex (19, 20). There is a theory that the excitation of unilateral cortical regions leads to suppression of the contralateral side, so the high-frequency stimulation on the left can achieve the same therapeutic effect in the right position by low frequency (21). It is consistent with the results observed by Yuan et al. that no matter high-frequency rTMS at left PFC or low-frequency rTMS at right PFC effectively reduced craving rate for MA abusers (22). Whether the patient requires two different and separate treatment protocols for drug seeking and impulsivity is yet to be understood.

The study is limited by a short period of treatment and follow-up time window. It is still necessary to understand the lasting effects of rTMS on impulsivity improvement; it is also important to examine if rTMS also improves risk decision making, which partly relies on behavioral inhibition ability. In addition, it will be interesting to understand if targeting motor cortical areas (e.g., SMA, M1) would also modulate the motor impulsivity and provide a full picture of impulsivity treatment in substances of abuse.

Taken together, the recent study by Yuan et al. opened novel possibility of impulsive behavior intervention for drug dependence and potentially imply on relapse behavior. Neuroimaging studies are required to elucidate the underlying neuroplasticity and circuitry changes following rTMS treatment.

## Author Contributions

HW, CY, and XL contributed equally to this work. HW and CY drafted the manuscript. XL revised the manuscript. WL and FH provided funds. All authors contributed to the article and approved the submitted version.

## Funding

The study was supported by the Medical Science and Technology Project in Ningbo (2017A54 and 2018A31), Science and Technology Project in Yongkang (201634), and Ningbo Municipal Innovation Team of Life Science and Health (2015C110026).

## Conflict of Interest

The authors declare that the research was conducted in the absence of any commercial or financial relationships that could be construed as a potential conflict of interest.
